# Development and Evaluation of Compact Semi-Synthetic Promoters for Enhanced Antigen Expression in Adenoviral-Vectored Vaccines

**DOI:** 10.3390/vaccines14030260

**Published:** 2026-03-13

**Authors:** Matěj Hlaváč, Susan J. Morris, Barbara Dema, Marta Ulaszewska, Zakia Al-Hareth, Bruno Douradinha, Sarah C. Gilbert

**Affiliations:** 1 Pandemic Sciences Institute, Nuffield Department of Medicine, University of Oxford, Oxford OX3 7DQ, UK; 2 CAMS Oxford Institute, Chinese Academy of Medical Sciences & Peking Union Medical College, University of Oxford, Oxford OX3 7BN, UK

**Keywords:** adenoviral-vector vaccines, ChAdOx1, synthetic promoters, antigen expression

## Abstract

Background/Objectives: The large size of commonly used regulatory elements such as the cytomegalovirus (CMV) immediate-early promoter imposes a significant burden on the already restricted payload capacity of first-generation adenoviral vectors, potentially hindering the development of multi-antigen vaccine candidates. To address this limitation, we have engineered a panel of novel, small, semi-synthetic promoters designed to leverage the changes in transcriptomic milieu following adenoviral vector entry. Methods: Eight synthetic enhancer modules (SE1–SE8) were designed in silico, each composed of transcription factor binding sites (TFBSs) previously found in host genes that are upregulated during early adenoviral infection. These synthetic enhancers were coupled with a minimal CMV core promoter to generate a panel of compact semi-synthetic promoters (cSE1–cSE8), and their activity was evaluated in the context of ChAdOx1 viral vectors expressing GFP or a modified *Plasmodium falciparum* circumsporozoite (CSN) antigen. Promoter performance was characterised in vitro via flow cytometry, RT-qPCR, and Western blotting, and in vivo by quantifying antigen-specific T-cell (IFN-γ ELISpot) and IgG antibody (ELISA) responses in BALB/c mice. Results: In vitro characterisation revealed a wide range of promoter activity across the panel, with cSE3 and cSE5 driving transgene expression levels comparable to the benchmark CMV promoters despite their markedly reduced genomic footprint. In vivo, ChAdOx1 vectors incorporating cSE3 and cSE5 elicited potent antigen-specific T-cell and IgG responses that were comparable to those induced by the larger CMV control promoters. Conclusions: We have successfully developed semi-synthetic promoters that match the potency of the much larger, frequently used CMV promoters whilst simultaneously reducing genomic footprint. These novel regulatory elements will facilitate the design of next-generation vaccines, particularly those requiring large antigens or multi-antigen cassettes.

## 1. Introduction

Adenoviral vectors represent a well-established platform for vaccine development, having been clinically validated across a broad range of indications [1]. For first generation vectors, the strategic removal of wild-type (WT) adenoviral regions, most notably E1 and E3 (ΔE1/ΔE3), serves two key purposes. First, the deletions enhance safety by rendering the vector replication-incompetent and eliminating its intrinsic immune-modulating functions. Second, genomic space is liberated, permitting insertion of a heterologous antigen expression cassette [2,3]. Since these modifications permit the rapid substitution of antigen-encoding sequences without further need to alter the underlying vector backbone, expedited development timelines can be achieved [1]. Furthermore, scalable manufacturing processes and a favorable cost-per-dose profile render adenoviral vector vaccines an ideal tool for addressing neglected tropical diseases in low-income settings and for rapid responses to emerging pathogens [2,3]. The seminal utility of this platform was demonstrated during the COVID-19 pandemic through the deployment of the chimpanzee adenovirus-derived vaccine ChAdOx1 nCoV-19 (developed as AZD1222; marketed as Vaxzevria^®^ and Covishield^®^). Developed on an unprecedented timescale, over three billion doses have been produced to date, highlighting the beneficial global impact of the technology [4].

Despite these many advantages, adenoviral vector platforms are constrained by the physical packaging limit of the viral capsid. The theoretical insert capacity for first-generation adenoviral vectors, which constitute the majority of clinically developed candidates, is often cited as approximately 8 kb [2]. While precise packaging limits are inherently serotype-specific, historical data derived largely from human adenovirus serotype 5 (HAdV-C5) indicate that vectors approaching 105% of the WT length are prone to genetic instability [5,6]. Although a definitive insert limit for chimpanzee-adenovirus Y25 (ChAdY25)-derived ChAdOx1 vectors has not been formally established, empirical evidence from large-scale manufacturing suggests a functional threshold closer to 7 kb; constructs exceeding this size are frequently associated with reduced rescue efficiency, low viral titres, and transgene instability (C. Powers, Viral Vector Core Facility, University of Oxford, personal communication). This finite insert capacity represents an important limitation that hinders the development of vaccines encoding large or multi-antigen expression cassettes within a single vector.

**Figure 1:**
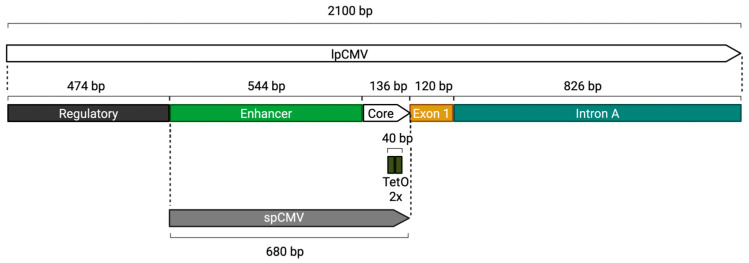
Composition of the lpCMV and spCMV promoters. The lpCMV promoter is 2100 bp in length and includes a regulatory region, enhancer, core promoter, exon 1, and Intron A. In contrast, the spCMV promoter is a truncated 680 bp version that retains the enhancer and core regions but lacks the regulatory region, Exon 1 and Intron A. Both promoter configurations include two tetracycline operator (TetO) sequences positioned downstream of the TATA box.

One way to address size constraints of the insert would be to reduce the footprint of the regulatory elements found within the antigen cassette. Beyond the bespoke antigen-coding sequence, cassettes contain a promoter to drive transgene expression and a 3′ UTR that houses the polyadenylation (polyA) signal [2,3]. The promoter is a key determinant of antigen expression magnitude and kinetics, both of which influence overall vaccine immunogenicity [7,8]. Historically, clinically validated adenoviral vector vaccines have relied on variants of the cytomegalovirus (CMV) immediate-early (IE) promoter. For adenoviral vector vaccine candidates developed at the University of Oxford, two specific configurations of the CMV promoter have predominated, termed the “long” and “short” CMV promoters (lpCMV and spCMV, respectively, Figure 1). The lpCMV is 2100 bp in length and encompasses the WT CMV major IE promoter-enhancer, exon 1, and the intron A sequence [9]. By contrast, the spCMV variant is only 680 bp; it retains the minimal core promoter and enhancer region but lacks the exon and intron sequences [9]. Published comparisons of both promoters utilising HAdV-C5-derived vectors encoding the *Plasmodium berghei* circumsporozoite protein (CS) demonstrated that lpCMV significantly enhanced antigen transcript abundance in vitro relative to spCMV, and improved CS-specific immune responses in mice [10]. Similarly, in ChAd63 vectors encoding a codon optimised version of the *Plasmodium falciparum* CS antigen (hereafter referred to as CSN, see results section for further details), the lpCMV promoter drove higher antigen expression in vitro and elicited antigen-specific cellular immune responses in mice that were approximately 1.4-fold higher than those observed with spCMV [9]. Consequently, lpCMV has been adopted as the default promoter for most vaccine development programmes, including the ChAdOx1 nCoV-19 vaccine [11]. However, the large size of lpCMV occupies a substantial fraction of the 7 Kb insert limit, meaning that spCMV can provide a useful alternative for larger antigen sequences. Unfortunately, even spCMV can act as a limitation for particularly large antigen sequences or for multivalent vectors where multiple antigen cassettes (and therefore multiple promoters) are required. Additionally, the presence of multiple sequence-identical promoters introduces the risk of recombination-mediated deletions and/or rearrangements within the vector genome.

Synthetic promoters potentially provide a compelling solution to these challenges. These regulatory elements mirror the basic architecture of eukaryotic promoters, consisting of a minimal or “core” promoter—which recruits RNA polymerase II—paired with engineered enhancer modules. The enhancer modules are typically composed of clustered transcription factor binding sites (TFBSs) arranged in defined or random patterns that can boost transcriptional output from the core promoter [12].Such promoters can be tailored for context specificity or defined expression kinetics and, crucially, can match or exceed the activity of larger, WT promoters while maintaining a markedly reduced genomic footprint [12,13].

Previous studies using HAdV-C5 and HAdV-C2 have shown that adenoviral infection precipitates a dynamic shift in the host cell transcriptomic milieu [14,15,16,17]. The physical process of capsid attachment, internalization, and endosomal escape triggers various cellular sensors such as integrins, TLR9, and cGAS [18], which in turn activate downstream signaling cascades to drive rapid upregulation of host genes associated with antiviral immunity, most notably cytokines, as well as regulators of apoptosis and the cell cycle [14,15,16,17]. In this study, we have identified TFBS motifs enriched within the promoter regions of these upregulated genes and used them to design a panel of synthetic enhancer modules. When coupled with a minimal CMV core promoter, these novel, compact semi-synthetic promoters demonstrated that leveraging the host response to vector entry allows for a significant reduction in promoter size without compromising transcriptional potency, transgene expression magnitude, or immunogenicity responses.

## 2. Materials and Methods

### 2.1. TFBS Identification and In Silico Design of Synthetic Enhancer Modules

**Figure 2:**
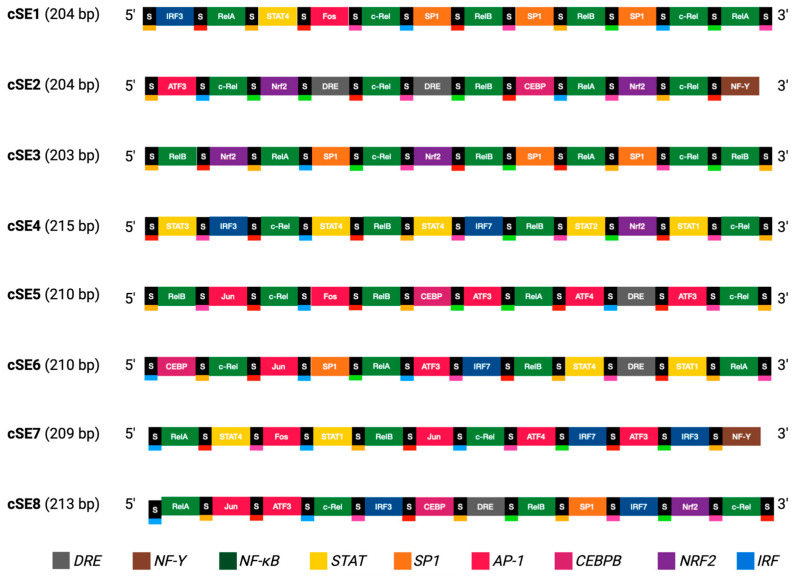
Composition of the novel synthetic enhancer units. The diagram depicts the arrangement of transcription factor binding motifs and spacer sequences that together form each individual synthetic enhancer module. Binding motifs, which constitute the fundamental functional elements of the enhancers, are colour-coded according to their associated transcription factor families. The five distinct spacer sequences used in the construction of the enhancer units are also indicated, each in a distinct colour.

The promoter regions from genes upregulated during the early stages of infection by different adenoviruses (Table 1), as described elsewhere [14,15,16,17], were obtained from the Eukaryotic Promoter Database [19]. These files, containing sequences spanning 500 bp upstream and 100 bp downstream of the transcription start sites (TSSs), were analysed for TFBSs using the CiiiDER computational toolkit for transcription factor binding analysis [20]. The JASPAR2020 vertebrate TFBS position frequency matrices (PFMs) dataset served as the reference library [21]. A confidence cut-off of 0.1 was set to ensure rigorous scanning. Based on this analysis, several TFBSs were selected to form the building blocks for eight novel synthetic enhancer (SE) modules (Figure 2, Table 2). Each SE consists of 12 TFBSs, each separated by one of five 6 bp-long spacer sequences (5′ACTAGA 3′, 5′TATCCT 3′, 5′ACAGAT 3′, 5′ACCTTA 3′, 5′AAAGGT 3′), which were used to enhance sequence heterogeneity and prevent steric obstruction from multiple transcription factor (TF) binding in short proximity. The number of identical building blocks within each enhancer was limited to three to reduce TF titration, and overall effects on the target cells’ transcriptome. Unless part of a binding motif, CpG dinucleotides were removed where possible to reduce silencing by methylation. The arrangement of building blocks and spacer sequences was optimised to limit internal repeats and prevent recombination; no intra-enhancer repeats >15 bp and inter-enhancer repeats were limited to <30 bp to potentiate the use of multiple different enhancers within the same vector. Finalised SE sequences (Table S1) were analysed using CiiiDER software (http://www.ciiider.org accessed on 4 March 2026) to exclude introduction of novel, undesired TFBS sequences at the TFBS-spacer junctions.

**Table 1:** Genes upregulated in early AdV infection ^1^.

Group	Genes
Cytokines	*CXCL1, IL6, CCL2, CCL20, CXCL3, TNFSF15, IL1B, HGF, IL11, CXCL10, RALA, FGF2, FGF7, GDF15, AREG, HGF*
Cell cycle regulation	*CDKN1A, FYN, PLK2, AHR, RGCC*
Apoptosis	*MDM2, TNFAIP2, TNFAIP3, TNFAIP6, TNFAIP8, TNFSF15, BIRC3*
Transcription factors	*ATF3, ATF4, CEBPB, NR4A1*

^1^ Based on transcriptomics data from HAdV-C2 and HAdV-C5 infected cells. Combined from [14,15,16,17].

**Table 2:** TFBS building blocks used in the design of the synthetic enhancers.

TFBS	Sequence 5′-3′ ^1^	Number Per Enhancer Module:							
		SE1	SE2	SE3	SE4	SE5	SE6	SE7	SE8
c-Rel	GGGGATTTCC	2	3	2	2	2	1	1	2
RelA	GGGAATTTCC	2	1	2	-	1	2	1	1
RelB	GAATTCCCC	2	1	3	2	2	1	1	1
SP1	GGGGCGGGGT	3	-	3	-	-	1	-	1
NF-Y	CAGCCAATCA	-	1	-	-	-	-	1	-
Jun	ATGAGTCAT	-	-	-	-	1	1	1	1
Fos	TGTGACTCAT	1	-	-	-	1	-	1	-
ATF3	GATGACGTCATC	-	1	-	-	2	1	1	1
ATF4	ATGATGCAATA	-	-	-	-	1	-	1	-
CEBPB	ATTGCACAAT	-	1	-	-	1	1	-	1
IRF3	GAAACGGAAACCGAAAC	1	-	-	1	-	-	1	1
IRF7	CGAAAGTGAAAGT	-	-	-	1	-	1	1	1
STAT1	GGAAAATGAAACTG	-	-	-	1	-	1	1	-
STAT2	AGAAACAGAAA	-	-	-	1	-	-	-	-
STAT3	AGAAACAGAAA	-	-	-	1	-	-	-	-
STAT4	TTTCCAGGAAA	1	-	-	2	-	1	1	-
Nrf2	ATGACTCAGCA	-	2	2	1	-	-	-	1
DRE	GCTTGCGTGAGAAG	-	2	-	-	1	1	-	1

^1^ Exact sequences of the binding motifs worked out from the position frequency matrices, available from the JASPAR open access database of transcription factor binding sites [21].

### 2.2. Construction of Semi-Synthetic Promoters

The SE sequences were ordered from and synthesised by GeneArt (Thermo Fisher Scientific, Regensburg, Germany). The 136 bp “core” promoter region was derived from the minimal sequence of the lpCMV promoter, which was described previously [10]. DNA fragments corresponding to the synthetic enhancers and the CMV core promoter were amplified via PCR and assembled upstream of the Enhanced Green Fluorescent Protein (EGFP) reporter gene open reading frame (ORF) and the bovine growth hormone (bGH) polyadenylation signal. The complete expression cassettes were cloned into a Gateway^®^-compatible pENTR™ plasmid using the NEBuilder^®^ HiFi DNA Assembly Master Mix (New England Biolabs, Ipswich, MA, USA). The combination of the CMV core promoter with the individual SE sequences generated a library of eight semi-synthetic promoters, designated cSE1–cSE8. The sequence integrity of all constructs was confirmed by Sanger sequencing prior to downstream application.

### 2.3. Plate-Based GFP Assays

HeLa and A549 cells were obtained from the American Type Culture Collection (ATCC, Manassas, VA, USA), while Huh7 cells were provided by the Jenner Institute (Oxford, UK). All cell lines were maintained in Dulbecco’s Modified Eagle Medium (DMEM) supplemented with 10% foetal bovine serum (FBS), 2 mM L-glutamine, and penicillin-streptomycin (all from Thermo Fisher Scientific, Waltham, MA, USA). For promoter activity assays, cells were seeded at 1 × 10^5^ cells per well in 24-well plates 24 h prior to transfection. Cells were transfected with 300 ng of plasmid DNA using Lipofectamine^®^ 2000 (Thermo Fisher Scientific) at a 1:3 DNA-to-reagent ratio. At 24h post-transfection, cells were harvested into 300 µL PBS, lysed via two freeze–thaw cycles, and clarified by centrifugation (16,000× *g*, 5 min). GFP fluorescence was quantified in black 96-well MaxiSorp™ immuno plates (Thermo Fisher Scientific) using a CLARIOstar Plus microplate reader (BMG Labtech, Ortenberg, Germany), and concentrations were determined by interpolation against a recombinant GFP (Millipore, Burlington, MA, USA) standard curve.

### 2.4. Generation of Recombinant ChAdOx1 Vectors

Expression cassettes, each containing a semi-synthetic promoter, the EGFP ORF, and the bGH polyA signal, and flanked by attL recombination sites within the aforementioned pENTR plasmids, were mobilised into the ChAdOx1 genome. Recombination was performed into an existing ChAdOx1 bacterial artificial chromosome (BAC) destination vector containing the corresponding attR sites at the E1 locus [22], using the Gateway^®^ LR Clonase™ II Enzyme Mix (Invitrogen, Waltham, MA, USA). This generated a panel of eight new ChAdOx1-GFP constructs. Subsequently, Gateway^®^ entry plasmids encoding a codon-optimised version of the *P. falciparum* circumsporozoite protein (CSN) were constructed. The CSN ORF was excised from an existing entry vector as described elsewhere [9], and cloned into each of the pENTR-promoter plasmids, replacing the GFP reporter gene downstream of the novel promoters. ChAdOx1-CSN vectors were generated as described above. For both GFP and CSN, constructs driven by the previously described lpCMV and spCMV promoters [9] were generated in parallel to serve as positive controls. ChAdOx1-GFP vectors were rescued, amplified, and purified in-house using T-REx™-293 cells according to established protocols [22]. The ChAdOx1-CSN vaccine vectors were manufactured by the Viral Vector Core Facility (Pandemic Sciences Institute, University of Oxford). For all constructs, viral titres were determined via a standardised adenovirus hexon immunotitration assay, providing final virus concentrations in infectious units per millilitre (IU/mL) [22].

### 2.5. Flow Cytometry

Twenty-four-well plates were seeded with A549 cells at 1 × 10^5^ cells in 500 μL of DMEM per well and grown for 24 h. Cells were infected with ChAdOx1-GFP vectors at multiplicities of infection (MOIs) of 10 and 1. After 48 h, A549 cells were harvested and fixed with 8% paraformaldehyde (Thermo Fisher Scientific), followed by staining with the LIVE/DEAD Fixable Aqua Dead Cell Stain Kit (Invitrogen) according to the supplier’s instructions. Fluorescence readouts were acquired using the LSRFortessa Cell Analyzer (BD Biosciences, Franklin Lakes, NJ, USA). Data analysis was performed using FlowJo v10.8 (BD Biosciences).

### 2.6. Quantification of Antigen Expression via RT-qPCR

A549 cells were infected with ChAdOx1-CSN vectors at an MOI of 50. Cells were harvested 24 h post-infection, and total RNA was isolated using the RNeasy Mini Kit (Qiagen, Hilden, Germany) according to the manufacturer’s instructions. RNA concentrations were normalized prior to analysis to ensure equivalent input across samples. Quantitative reverse transcription PCR (RT-qPCR) was performed using the Luna^®^ Universal Probe One-Step RT-qPCR Kit (New England Biolabs, Ipswich, MA, USA) in 20 µL reaction volumes. Each reaction contained 1× Luna Universal Probe Master Mix, 1× WarmStart^®^ RT Enzyme Mix, 0.4 µM forward and reverse primers, 0.2 µM hydrolysis probe, and 100 ng of RNA template. All reactions were conducted in technical triplicates in optical 96-well plates on a QuantStudio 3 Real-Time PCR System (Applied Biosystems, Waltham, MA, USA). Custom primers and probes were synthesized by Integrated DNA Technologies (IDT, Coralville, IA, USA). The CSN antigen transcripts were detected using a primer-probe set targeting the bGH polyA signal, which is shared by all constructs: forward primer 5′-CTAAATGCTAGAGCTCGCTGAT-3′, reverse primer 5′-TAGGAAAGGACAGTGGGAGT-3′, and hydrolysis probe 5′-FAM-AGTTGCCAGCCATCTGTTGTTTGC-TAMRA-3′. The adenoviral DNA-binding protein (DBP) transcript levels were quantified to ensure equivalent viral input due to variability of the hexon immunostaining assay using forward primer 5′-GATGATGGTGAAGAACCCTATGA-3′, reverse primer 5′-CATCAGTTGGAAGTTGGCTTTC-3′, and probe 5′-VIC-CCTGGGAGAAGGGCATGGAAATCA-MGB-3′. Endogenous gene expression was monitored using a commercially available TaqMan™ Assay for GAPDH (Cat. #4352934T; Life Technologies, Carlsbad, CA, USA) to control for RNA input consistency. Cycling conditions consisted of reverse transcription (55 °C, 10 min) and initial denaturation (95 °C, 1 min), followed by 40 cycles of denaturation (95 °C, 10 s) and annealing/extension (60 °C, 30 s). Absolute quantification was performed by interpolation against a standard curve generated from a seven-point, 10-fold serial dilution of linearized ChAdOx1-CS plasmid DNA (20 ng to 20 fg). Data were analysed using Design and Analysis Software v2.3 (Applied Biosystems).

### 2.7. Quantification of Antigen Expression via Western Blot

A549 cells were infected with ChAdOx1-CSN vectors at an MOI of 50 and harvested 24 h post-infection. Cell pellets were lysed in RIPA Lysis and Extraction Buffer supplemented with Halt™ Protease Inhibitor Cocktail (Thermo Fisher Scientific). Protein concentrations were determined using the Pierce™ BCA Protein Assay Kit (Thermo Fisher Scientific), and lysates were normalised to ensure equivalent total protein loading. Samples were denatured in Laemmli Sample Buffer (Bio-Rad, Hercules, CA, USA) containing 100 mM dithiothreitol (DTT) at 95 °C for 10 min. Proteins were resolved on 4–15% Mini-PROTEAN^®^ TGX™ precast gels (Bio-Rad) and transferred to PVDF membranes using the Trans-Blot^®^ Turbo™ Transfer System (Bio-Rad). Immunodetection was performed using the iBind™ Western Device (Invitrogen) to automate blocking and antibody incubation steps. The CSN antigen was detected using the mouse monoclonal antibody 2A10 (1:1000; Malaria Research and Reference Reagent Resource Centre, Manassas, VA, USA). To control for viral transduction efficiency, the adenoviral DNA-binding protein (DBP) was detected using a rabbit polyclonal antibody (1:5000; Cat. #CSB-PA365892ZA01HIL, CUSABIO, Houston, TX, USA). Primary antibodies were detected using HRP-conjugated secondary antibodies: goat anti-mouse IgG (1:5000; Cat. #31430, Invitrogen) for CSN and goat anti-rabbit IgG (1:10,000; Cat. #31460, Invitrogen) for DBP. Blots were developed using Clarity™ Western ECL Substrate (Bio-Rad) and imaged on a ChemiDoc™ MP Imaging System (Bio-Rad). Band intensities were quantified using ImageJ software (https://imagej.net/ij/ accessed on 4 March 2026, NIH, Bethesda, MD, USA). Target protein density was normalised to DBP density to calculate relative protein abundance.

### 2.8. Mouse Immunisations

Inbred female BALB/cOlaHsd mice, aged 6 to 8 weeks, were purchased from Envigo RMS (Inotiv) Ltd. (Cambridgeshire, UK). Mice were housed in individually ventilated cages (IVCs) under specific pathogen-free (SPF) conditions. The housing environment maintained a constant temperature and humidity, with a 13:11 h light-dark cycle (7 a.m. to 8 p.m.). After a week of acclimatation, mice were randomly allocated into groups of 5 and immunised intramuscularly with 1 × 10^8^ infectious units (IUs) of either ChAdOx1-cSE3 CSN, ChAdOx1-cSE5 CSN, ChAdOx1-cSE4 CSN, ChAdOx1-spCMV CSN or ChAdOx1-lpCMV CSN in a total volume of 50 μL, which was injected into the musculus tibialis of the left hind leg. The dose was selected based on previously established protocols [9] as it typically provides an optimal balance between inducing high cellular and humoral immune responses while maintaining a favorable safety and reactogenicity profile in mice. Mice experiments were performed in accordance with the UK Animals (Scientific Procedures) Act under project license number PP2352929, granted by the UK Home Office following an ethical review by the University of Oxford Animal Welfare and Ethical Review Board (AWERB). These studies adhered to the Animal Research: Reporting of In Vivo Experiments (ARRIVE) guidelines [23] following the principles of the 3Rs (replacement, reduction and refinement). For the induction of short-term anaesthesia, animals were anaesthetised using vaporised IsoFlo^®^ (Zoetis UK Limited, Surrey, UK). All animals were humanely sacrificed at the conclusion of each experiment following an approved Schedule 1 procedure and organs were harvested and processed in BSL2 cabinets.

### 2.9. Immunogenicity Assessment

CSN-specific cellular responses were quantified using an ELIspot assay. Peripheral blood mononuclear cells (PBMCs) were isolated from blood samples drawn two weeks post-vaccination and from terminal bleeds collected at the four-week endpoint. Following the protocol described by Sridhar et al. [10], cells were restimulated ex vivo using a pool of synthetic overlapping oligopeptides spanning the entire CSN protein sequence. Blood sera collected four weeks post-immunisation were evaluated for CSN-specific IgG using ELISA following previously established protocols [24], with titres expressed in ELISA units (EU) relative to a reference standard derived from a CS-specific monoclonal antibody (2A10).

### 2.10. Statistical Analysis

Statistical analyses and data visualisation were performed using GraphPad Prism version 10.6.1 (799) for macOS (GraphPad Software, San Diego, CA, USA). Unless otherwise indicated, grouped data are presented as the mean ± standard error of the mean (SEM). The choice of statistical test was determined by the data distribution and is explicitly stated in the results section for each experiment. Generally, normally distributed datasets were analysed using One-way ANOVA, with multiple comparisons corrected using the Holm–Šídak method. For data that did not follow a normal distribution, the Kruskal–Wallis test was used, followed by Dunn’s test for multiple comparisons. For animal experiments, cohort sizes were determined a priori in accordance with the 3Rs principle of Reduction, and statistical power calculations were performed using effect size estimates based on previously published data [9].

## 3. Results

### 3.1. Design of Semi-Synthetic Promoters

To identify transcriptional regulators capable of enhancing promoter activity within the context of adenoviral-vectored vaccines, CiiiDER software, which predicts and analyses transcription factor binding sites, was used to identify transcriptional regulators capable of enhancing promoter activity following adenoviral vector infection [14,15,16,17]. Specifically, the analysis was restricted to genes elevated within the early hours post-infection (Table 1) to capture the peak innate signalling period and bypass the confounding effects of viral early gene expression, which typically subverts host cell signalling [18]. While the source transcriptomic studies utilised WT adenovirus, this early window is particularly relevant for the E1/E3-deleted vectors used in this study. This is because the initial triggers of the host response, encompassing integrin binding, endosomal escape, and the sensing of the viral genome, are mechanistically identical during the entry phase, occurring independently of viral gene synthesis [18]. Consequently, the transcription factor landscape induced during the early hours of infection remains consistent across both wild-type and first-generation vectors. From this analysis, a series of consensus TFBS motifs were identified and further selected based on the established functions of the corresponding transcription factor families in gene expression regulation. Binding motifs for the NF-κB (c-Rel, RelA, RelB) and AP-1 (Jun, Fos) families were chosen to leverage the inflammatory and stress cascades initiated by viral particle detection via cellular integrins and pattern recognition receptors [18]. Similarly, IRF (IRF3, IRF7) and STAT motifs were included to exploit the host’s immediate antiviral interferon response, which is typically triggered by cytosolic sensors like the cGAS-STING pathway upon detection of the adenoviral DNA genome [18]. Binding motifs for nuclear factor Y (NF-Y), CCAAT/enhancer-binding protein beta (CEBPB), and specificity protein 1 (SP1) were also included, alongside Nuclear factor E2-related factor 2 (Nrf2) and the dioxin-response element (DRE) due to their established roles in basal and innate immunity signalling [25,26]. Motifs with poorly characterised regulatory effects were excluded to prevent potential attenuation of enhancer activity. All TFBS sequences were derived from position frequency matrices obtained from the JASPAR database [21].

The selected TFBSs served as the building blocks for the design of eight synthetic enhancers (SE1–SE8; Figure 2, Table 2 and Supplementary Table S1). Each enhancer was composed of twelve TFBS motifs separated by 6 bp spacer sequences, included to prevent potential steric hindrance affecting transcription factor binding. The number of motifs was empirically selected to balance the requirement for high transcriptional potency with the compact size requirements necessitated by the limited payload capacity of the vector. Furthermore, the design ensured that sequence identity between the eight enhancers was minimised (avoiding homologous stretches > 30 bp) to limit the potential risk of homologous recombination should multiple promoters be utilised within a single vector.

Each enhancer was subsequently paired with a 136 bp minimal CMV core promoter to generate a library of novel semi-synthetic promoters, cSE1–cSE8. These constructs were cloned upstream of a *EGFP* reporter gene ORF and a bGH polyA signal within a Gateway^®^-compatible entry plasmid for downstream characterisation.

### 3.2. Initial Promoter Evaluation

The transcriptional potency of the semi-synthetic promoter library was first evaluated in a plasmid context via transient transfection, in the absence of adenovirus. Plasmid constructs expressing EGFP under the control of each semi-synthetic promoter (cSE1–cSE8) were transfected into three distinct cell lines; A549, HeLa, and Huh7. To assess the specific contribution of the synthetic enhancers to transcriptional activity, a negative control construct containing only the minimal 136 bp CMV core promoter, common to all SE (and both spCMV and lpCMV) promoters, was included to establish baseline expression levels in the absence of an enhancer. Additionally, constructs incorporating spCMV and lpCMV were included as positive controls to allow for a direct comparison of novel semi-synthetic promoters against established regulatory elements commonly utilised in clinically validated adenoviral vector vaccines. GFP concentration was quantified from cell lysates 24 h post-transfection to determine relative transcriptional activity.

**Figure 3:**
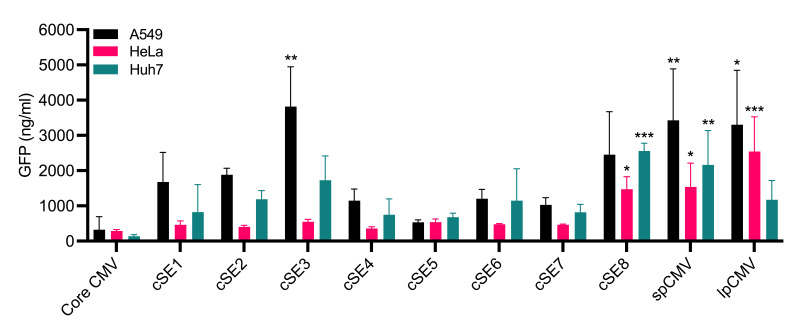
Comparative analysis of semi-synthetic promoter activity in human cell lines. A549, HeLa, and Huh7 cells were transfected with plasmid constructs encoding GFP driven by the indicated promoters. GFP concentration was quantified 24 h post-transfection. Data are presented as the mean GFP concentration ± SEM (*n* = 3 independent experiments). The core CMV construct represents the basal promoter activity driven by the 136 bp minimal promoter without enhancer elements. Statistical significance was determined using a one-way ANOVA with Holm-Šídák correction for multiple comparisons. Asterisks indicate expression levels significantly greater than the baseline core CMV construct (* *p* < 0.05, ** *p* < 0.01, *** *p* < 0.001).

The initial screening revealed distinct cell line-dependent expression from each promoter (Figure 3). In A549 cells, the cSE3 promoter emerged as the most potent semi-synthetic candidate, driving a mean GFP concentration approximately 12-fold higher than the enhancer-less CMV core baseline (3816 ± 652 vs. 320 ± 215 ng/mL; *p* < 0.01). Notably, the activity of cSE3 was statistically indistinguishable from that of the industry-standard benchmarks, achieving expression levels comparable to both spCMV (3427 ± 844 ng/mL) and lpCMV (3304 ± 891 ng/mL) controls (*p* > 0.05 for both comparisons). cSE8 also demonstrated robust activity (2454 ± 704 ng/mL). Across the SE panel, cSE5 drove the lowest expression levels (533 ± 39 ng/mL), significantly lower than both spCMV and lpCMV benchmarks (*p* < 0.05).

In Huh7 cells, the hierarchy of promoter potency shifted. cSE8 was the highest-performing synthetic construct, achieving a mean expression level ~18-fold over the enhancer-less core CMV promoter (2557 ± 128 vs. 139 ± 24 ng/mL; *p* < 0.001), and significantly higher than cSE1, 4, 5, and 7 (*p* < 0.05). In this context, the activity of cSE8 was statistically equivalent to both spCMV (2163 ± 562 ng/mL) and lpCMV (1172 ± 316 ng/mL, *p* > 0.05 for both comparisons). cSE3 remained a significant driver of expression (1729 ± 399 ng/mL), whereas cSE5 was again the lowest-ranking synthetic variant (677 ± 66 ng/mL).

Finally, in HeLa cells, the overall efficacy of the semi-synthetic promoters was markedly reduced. Here, the lpCMV promoter was the dominant driver (2535 ± 571 ng/mL). Among the synthetic library, cSE8 (1467 ± 204 ng/mL) achieved the highest expression. The lowest-performing candidate was cSE4 (350 ± 31 ng/mL), which performed close to the Core CMV baseline (284 ± 22 ng/mL; *p* > 0.05).

The minimal Core CMV promoter consistently drove the lowest expression across all cell lines, as expected. This confirmed that the transcriptional output of the cSE constructs was enhanced, to differing degrees, by the synthetic enhancer modules. Across all tested cell lines, cSE8 acted most comparably to spCMV and lpCMV.

### 3.3. Characterisation of Promoter Activity in ChAdOx1 Vector-Infected Cells

To evaluate the performance of the semi-synthetic promoters within their intended biological context—i.e., an intracellular environment featuring an altered gene expression profile resulting from adenoviral infection—the expression cassettes composed of each individual semi-synthetic promoter (cSE1-8), *EGFP* reporter gene ORF and a bGH polyA signal were subcloned into the ChAdOx1 vector platform at the E1 locus. For control purposes, vectors expressing GFP from lpCMV and spCMV were generated in parallel. Viruses were rescued and amplified in T-REx™-293 cells to generate a panel of matched ChAdOx1-GFP vectors differing only by the promoter sequence.

**Figure 4:**
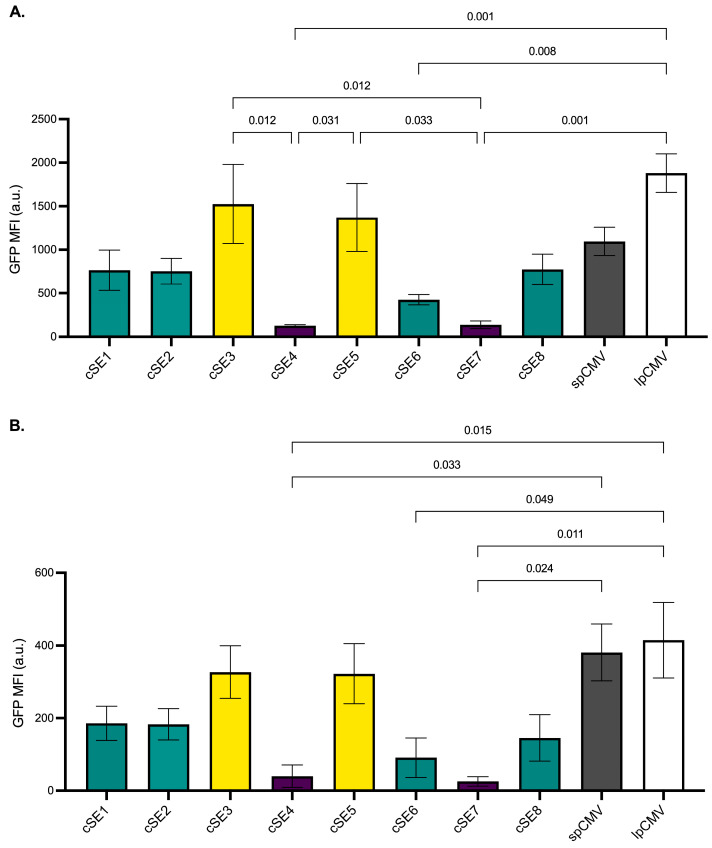
Flow cytometric analysis of promoter-driven GFP expression in A549 cells infected with ChAdOx1. Cells were infected with ChAdOx1-GFP vectors at an MOI of 10 (**A**) or 1 (**B**), and GFP expression was quantified 48 h post-infection. Data represent the mean fluorescence intensity (MFI) ± SEM (*n* = 3 independent experiments). cSE1–cSE8 denote semi-synthetic promoters; spCMV and lpCMV denote the short (683 bp) and long (2100 bp) CMV benchmark controls, respectively. Bars are colour-coded by transcriptional potency: high (yellow), moderate (teal), or low (purple), with CMV controls in gray (spCMV) and white (lpCMV). Statistical significance was assessed using one-way ANOVA with Holm-Šídák multiple comparisons test. Adjusted p-values are displayed; comparisons yielding *p* ≥ 0.05 are omitted.

A549 cells were infected with each vector at multiplicities of infection (MOIs) of 10 and 1 and analysed by flow cytometry 48 hrs post-infection, revealing a broad range of promoter activities across the panel. At MOI 10 (Figure 4A), ChAdOx1-GFP vectors employing cSE3 or cSE5 exhibited the highest mean fluorescent intensity (MFI) values (1525 ± 454 MFI and 1371 ± 391 MFI, respectively). Notably, the activity of these novel promoters was comparable to that observed with vectors utilising spCMV (1095 ± 161 MFI) and lpCMV (1880 ± 221 MFI, both *p* > 0.05). In sharp contrast, cSE4 and cSE7 displayed markedly reduced activity, yielding MFI values of 127 ± 13 and 137 ± 44 MFI, respectively. This represented a greater than 10-fold reduction in potency compared to the top performers. The remaining synthetic promoters (cSE1, cSE2, cSE6, and cSE8) exhibited intermediate activity, with FI values that ranged from 425 to 774.

The pattern of promoter activity was largely preserved at MOI 1 (Figure 4B), albeit with lower absolute fluorescence values, as expected. Again, constructs incorporating lpCMV (415 ± 104 MFI) and spCMV (381 ± 79 MFI) were matched by cSE3 (327 ± 73 MFI) and cSE5 (322 ± 83 MFI, *p* < 0.05 for both comparisons). Also consistent with the higher-MOI data, constructs containing the cSE4 and cSE7 promoters drove low expression levels relative to the rest of the panel (40 ± 31 and 25 ± 13 MFI, respectively).

Based on the flow cytometry data, the semi-synthetic promoter library was stratified into three functional categories: high activity (cSE3, cSE5), moderate activity (cSE1, cSE2, cSE6, cSE8), and low activity (cSE4, cSE7).

### 3.4. Transcript and Protein Expression Analysis of ChAdOx1-CSN Constructs

To determine whether the promoter activity hierarchy observed using the EGFP reporter translated to a more relevant antigen, we further tested their activities using the CSN antigen. CSN is a codon optimised version of the *P. falciparum* CS antigen that additionally lacks the C-terminal 9aa and has been described previously [9]. Based on the results from the ChAdOx1-GFP studies, three promoters were selected for further study: cSE3 and cSE5 were selected to represent the highest-performing promoters, and cSE4 was included as the lowest-performing candidate. As before, matched vectors containing spCMV or lpCMV were made and tested alongside for comparison.

**Figure 5:**
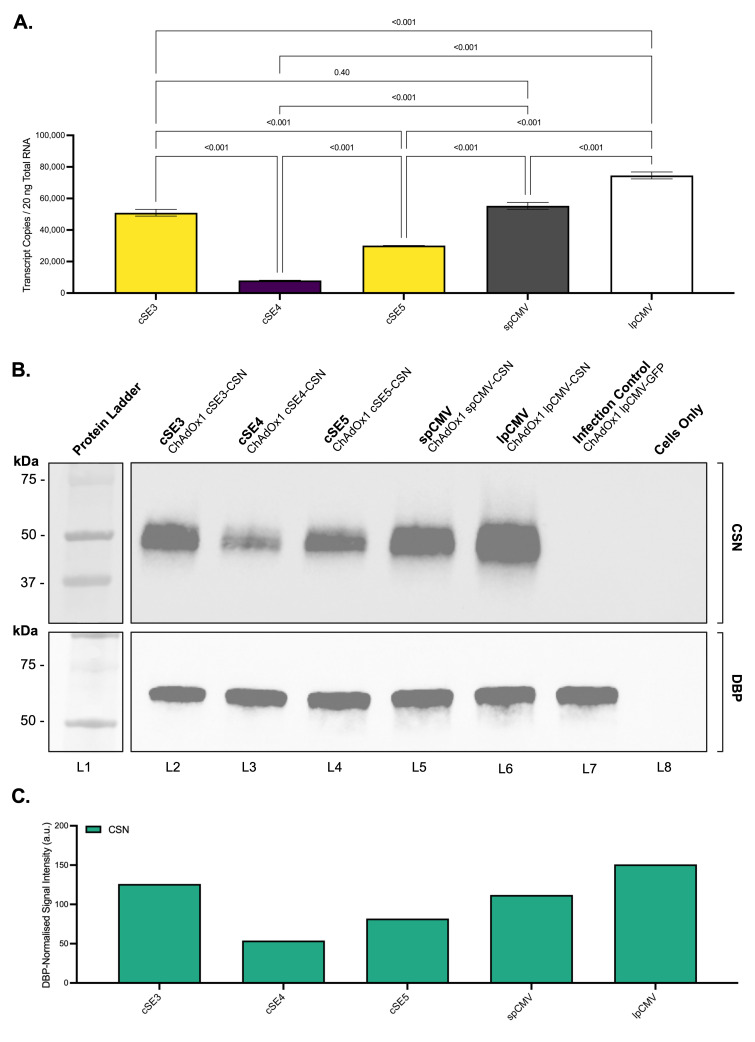
CSN transcript and protein abundance driven by different promoters from ChAdOx1 vectors. (**A**) CSN transcript levels in A549 cells. A549 cells were infected with ChAdOx1-CSN vectors (MOI 50) and harvested 24 h post-infection. The RNA was isolated from the cell lysates, and absolute CSN transcript copy number per 20 ng total RNA determined by one-step RT-qPCR targeting the 3′ UTR. Data are presented as mean ± SD (*n* = 3 technical replicates). Statistical significance was assessed using one-way ANOVA with Holm-Šídák multiple comparisons test. (**B**) Representative immunoblots of A549 lysates harvested 24 h post-infection. Top panel: CSN antigen (≈42 kDa). Bottom panel: DBP viral control (≈65 kDa). (**C**) Semi-quantitative densitometry performed using ImageJ. CSN signal intensity was normalized to DBP. Data represent relative protein expression levels expressed in arbitrary units (a.u.).

Firstly, the relative transcriptional activity of the selected promoters was quantified via RT-qPCR in A549 cells infected with each ChAdOx1-CSN vector at an MOI of 50. Analysis of CSN transcript abundance revealed marked differences among the promoters (Figure 5A). The lpCMV-containing vector yielded the highest transcript levels (mean ≈7.5 × 10^4^ copies per 20 ng RNA), significantly higher than all other constructs (*p* > 0.001 for all comparisons), followed by spCMV and cSE3 (≈5.5 × 10^4^ and 5.0 × 10^4^ copies, respectively). Statistical analysis confirmed that cSE3 activity was comparable to the spCMV benchmark (*p* = 0.40). cSE5 produced transcript levels that were significantly lower than the other promoters (≈3 × 10^4^, *p* > 0.001) except for cSE4, which was markedly less active and produced the lowest transcript levels (≈8.0 × 10^3^ copies).

To corroborate these findings at the translational level, CSN protein abundance was assessed by Western blot. Immunoblotting revealed clear antigen-specific bands at the expected molecular weight (≈42 kDa, Figure 5B). Densitometric quantification, normalised to the DBP loading control confirmed the hierarchy established by RT-qPCR (Figure 5C). The lpCMV vector drove the highest relative expression (151 a.u.), followed by cSE3 (126 a.u.) and spCMV benchmark (112 a.u.). cSE5 displayed intermediate potency (82 a.u.), and cSE4 promoter exhibited the lowest activity (54 a.u.), yielding CSN expression levels approximately 2.5-fold lower than cSE3 and spCMV and 2.8-fold lower than lpCMV.

### 3.5. Immunogenicity of ChAdOx1-CSN Constructs Utilising Semi-Synthetic Promoters

**Figure 6:**
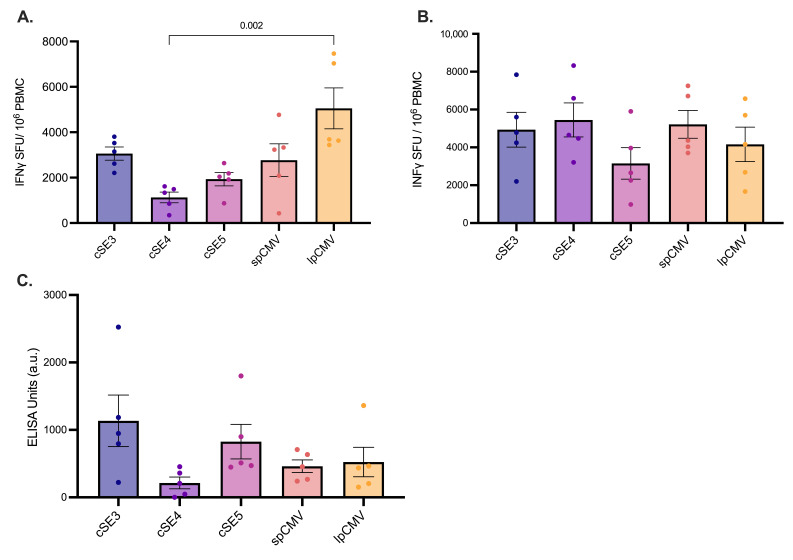
Immunogenicity of ChAdOx1 vectors expressing the *Plasmodium falciparum* CSN antigen in BALB/c mice. Female BALB/c mice (6–8 weeks old, *n* = 5 per group) were immunised intramuscularly with 1 × 10^8^ IU of ChAdOx1-CSN vectors. Antigen expression was driven by either semi-synthetic promoters (cSE3, cSE4, cSE5) or CMV-based controls (spCMV, lpCMV). Cellular immune responses were assessed by IFN-γ ELISpot following ex vivo stimulation with CSN peptide pools. Responses are expressed as spot-forming cells (SFCs) per 10^6^ input cells for PBMCs at day 14 (**A**) and day 28 (**B**) post-immunisation. (**C**) Humoral responses were measured at day 28 by CSN-specific IgG ELISA, with titres expressed in ELISA Units (EU). Each point represents an individual animal; horizontal lines indicate group means, and error bars represent the SEM. Statistical significance was assessed using the Kruskal–Wallis test with Dunn’s multiple comparisons test (**A**,**C**) or One-way ANOVA with Holm-Šídák multiple comparisons test (**B**). Exact p-values are shown.

Having confirmed CSN antigen expression from ChAdOx1 vectors incorporating the novel promoters, the immunogenicity of these constructs was evaluated. Female BALB/c mice were immunized with ChAdOx1-CSN vectors controlled by high-activity (cSE3, cSE5) or low-activity (cSE4) semi-synthetic promoters, alongside the spCMV and lpCMV controls. Cellular immune responses were assessed by IFN-γ ELISpot. At day 14 post-immunization (Figure 6A), the cellular responses across the groups closely mirrored the promoter activities that were observed in vitro. ChAdOx1 using lpCMV elicited the most potent cellular responses (mean ≈5000 SFC/10^6^ PBMCs), significantly higher than those elicited by cSE4 (*p* < 0.005) and showed a trend toward greater magnitude than cSE5 (*p* = 0.08). Among the semi-synthetic promoter candidates, cSE3 achieved the highest mean response (≈3000 SFC/10^6^ PBMCs), followed by cSE5 (≈2000 SFC/10^6^ PBMCs) and cSE4, which yielded a mean response of ≈1100 SFC/10^6^ PBMCs. At day 28, however, cellular responses had largely equalized across all experimental groups, maintaining mean frequencies in the range of ≈3000–5000 SFC/10^6^ PBMCs (Figure 6B). No statistically significant differences were detected between promoter constructs at this time point.

CSN-specific IgG responses were quantified by ELISA from sera drawn at day 28 post-immunisation (Figure 6C). cSE3 elicited the highest mean CSN-specific IgG titre (≈1100 EU), exceeding those elicited by cSE5 (≈800 EU) and cSE4 (≈200 EU). Notably, cSE3 and cSE5 performed with equal efficiency to lpCMV and spCMV (both ≈500 EU). Consistent with the cellular immunogenicity data, cSE4 underperformed relative to cSE3, with titres approximately five-fold lower, a difference that approached statistical significance (*p* = 0.059). In conclusion, cSE3 and cSE5 provoked CSN T-cell responses similar to levels observed with spCMV at day 14, and both spCMV and lpCMV by day 28, despite their vastly reduced size. They also induced antibody responses equivalent to the more commonly used promoters at day 28. Intriguingly, cSE4—included in the screen to represent weaker antigen expression—produced T-cell responses equivalent to the other promoters by day 28, despite the expected reduction in immunogenicity at day 14.

## 4. Discussion

The development of multi-antigen first generation adenoviral vector vaccines is hampered by the restricted payload capacity, a limitation caused by genomic packaging limits intrinsic to the vector platform itself. This study sought to address this limitation by demonstrating that the trade-off between promoter size and transcriptional potency can be effectively decoupled through the employment of synthetic enhancers.

Based on previous data [14,15,16,17], our synthetic enhancers were designed to bind transcription factors upregulated during the early stages of adenoviral infection, thereby providing a positive feedback effect on transgene expression. While the previous transcriptomic studies were performed on cells infected using human adenoviruses, we proceeded under the assumption that some or all the same genes would also be upregulated during ChAdOx1 infection. Our goal was to limit the size of our enhancers to ≈200 bp to keep them as compact as possible; this allowed us to use 12 TFBSs, inclusive of the 6 bp spacer sequences designed to separate each site and prevent steric hindrance that might arise from multiple TFs binding in close proximity. These considerations aligned with recommendations from a previous study on engineering synthetic enhancers [12]. While the spacers acted as an aid, limiting inter- and intra-enhancer homologous regions to <30 bp—necessary to prevent aberrant recombination events—proved especially challenging, and largely dictated TFBS order within each enhancer sequence. Fortunately, evidence exists to suggest that the relative position of each TFBS within an enhancer is largely irrelevant [13], except for NF-Y TFBS, which is commonly found 60–100 bp upstream of the TSS, and was thereby positioned accordingly [27].

The removal of the inter-enhancer homologies has the added benefit of rendering the system modular; when paired with different core promoters, multiple synthetic enhancers can be potentially used within a single vector, which would be especially useful for multivalent vaccines featuring more than one antigen cassette. This is particularly advantageous for the rational design of broadly protective vaccines against pathogens with high antigenic or lineage diversity, such as influenza virus, Ebola virus, and Lassa virus, where the stable co-expression of multiple distinct immunogens, or antigens from multiple viral strains, is required to achieve comprehensive coverage.

Here, we used the CMV core promoter, a decision based on the extensive validation of the parent promoter within the context of adenoviral-vectored vaccines, including ChAdOx1 [11,28,29]. Crucially, all semi-synthetic promoters described herein retain the Tet-operator (TetO) sequences upstream of the TATA region within the core promoter (Figure 1). This design feature ensures compatibility with the T-REx-293 producer cell line to repress antigen expression during production, thereby preventing any effects of the antigen on the health of the host cell during manufacturing and allowing a standardised manufacturing process to be employed.

Beyond adenoviral vectors, the underlying rationale of exploiting early innate immune and stress signaling cascades triggered by vector entry to rationally design synthetic enhancer modules holds translational potential for other platforms relying on heterologous promoters, such as Lentiviral, Herpes Simplex Virus (HSV), and Adeno-associated Virus (AAV) vectors. Replacing large, ubiquitous promoters in these systems with compact semi-synthetic alternatives would effectively reclaim valuable payload space, which is particularly critical for vectors with strict packaging limits.

While the synthetic enhancers were designed to leverage the specific transcription factor milieu induced by adenoviral infection, the initial evaluation of their activity followed transient transfection of naked plasmid DNA (Figure 3). Despite the absence of adenovirus in this initial screen, several of the eight semi-synthetic promoters drove increased GFP expression in one or more cell lines compared to the 136 bp minimal core promoter, which drove negligible expression in isolation. This enhancement is likely attributed to the synthetic enhancers responding to ubiquitously expressed transcription factors such as NF-κB and AP-1, which act as central mediators of general cellular stress and inflammatory responses. Also likely is that the physical process of transfecting high plasmid copy numbers triggers broad activation of the enhancers via transcription factor upregulation, as previously observed with IRF and STAT family members [30].

Introducing the semi-synthetic promoters into ChAdOx1 and infecting A549 cells re-stratified the promoter ranking compared to what was observed using plasmids alone and was deemed a more physiologically relevant assessment of their activity. Notably, cSE5, which performed poorly in the context of a transfected plasmid, exhibited markedly improved activity, while cSE8 potency declined compared to the plasmid data (Figure 3 vs. Figure 4). This shift suggested that changes in the specific intracellular signaling environment driven by vector entry—rather than generic transfection stress—were required to fully realise the activity of these promoters and revealed cSE3 and cSE5 as the lead candidate promoters for further study. ChAdOx1 vectors harbouring the “long” intron-A-containing CMV promoter consistently outperformed the “short” intronless version, which aligns with previous data for adenoviral vectors [9,10]. However, cSE3 and cSE5 matched the activity of both promoters in this assay at both tested MOIs, despite their markedly reduced size. Furthermore, the spectrum of activity observed across the semi-synthetic promoter panel highlights the utility of this series as a modular toolkit for fine-tuning gene expression. This is particularly advantageous for complex vector designs where maximal expression is not always desirable; for example, when co-expressing antigens and molecular adjuvants where stoichiometric control is required to balance efficacy with toxicity.

The vaccine antigen CSN is a modified version of *P. falciparum* CS, which in earlier studies required the use of the long CMV promoter to obtain maximum gene expression and immunogenicity [9]. Three of the novel promoters which had exhibited high (cSE3 and cSE5) or low (cSE4) activity were then tested for their ability to express this antigen. Data from ChAdOx1-CSN-infected cells largely mirrored that observed with ChAdOx1-GFP, although the difference between cSE3 and cSE5 became more obvious, with cSE3 proving the more potent promoter (Figure 4 vs. Figure 6). The enhanced protein expression from cSE3 was directly attributable to a greater amount of CSN transcript, and transcript/protein levels were comparable to those seen with the commonly used spCMV sequence. Importantly, cSE3-driven CSN expression elicited cellular and humoral responses in mice that were indistinguishable from those observed with the spCMV and lpCMV benchmarks at days 14 and 28. This result is encouraging and demonstrates that for this one antigen at least, the smaller cSE3 promoter (~350 bp) elicits immune responses comparable to those seen for the significantly larger spCMV (683 bp) and lpCMV (2100 bp) sequences.

While cSE4 induced cellular responses in mice that were comparably lower than those observed for cSE5 and cSE3 at day 14, as might be expected given its lower transcriptional activity during in vitro assessment, by day 28, the immunogenicity reached levels equivalent to those of the other vectors. One limitation of this study is that the kinetics of each promoter remain unknown, though they are likely rooted in the TFBS compositions of the different synthetic enhancer modules. It is possible that cSE4 activity ramps up slowly but becomes more potent over an extended time span compared to cSE3, perhaps in response to transcription factors that upregulate later and bind to cSE4-specific TFBSs that are lacking in cSE3. Future studies will aim to address the kinetics of these promoters during adenoviral infection and could potentially enable the engineering of vectors that express multiple antigens in a temporal manner.

A similar phenomenon was described by Chen et al., who compared the kinetics of luciferase expression in muscle tissue from matched adenoviral vectors driven by either Rous Sarcoma Virus (RSV) or CMV promoter in a murine model [8]. While the CMV promoter exhibited high initial expression that subsequently declined, RSV-driven expression increased over time, reaching equivalence between days 10–14 and surpassing CMV levels by approximately 10-fold by day 21 post-administration [8]. However, the impact of different promoter-mediated antigen expression kinetics profiles on the orchestration of cellular and humoral immunity following vaccination with adenoviral vectors currently remains poorly understood. Although Chen et al. did not evaluate immunogenicity [8], our results appear to mirror the protracted “slow-burn” kinetic profile the authors observed with the RSV promoter. The initial high-level antigen burst from cSE3 and CMV promoter variants likely facilitated the rapid T-cell priming observed at day 14. Conversely, the hypothesised delayed ramp-up of cSE4 may have allowed the cellular response to converge with the other vectors by day 28. However, the observation that cSE4-driven antibody titers at day 28 remained markedly lower compared to the remaining promoter constructs suggests that an early, high-intensity antigenic stimulus may be requisite for the efficient induction of the humoral response.

In addition to further validating the promoter panel in primary human cells and expanding the repertoire of core promoters to reduce the risks of homologous recombination in multi-cassette systems, future studies will aim to better characterise the in vivo expression kinetics of the novel promoters and elucidate the corresponding effects on the immune responses. Furthermore, the use of additional animal models, incorporating prime-boost schedules and detailed immunophenotyping will be valuable to further characterise the qualitative immune profiles driven by vaccine constructs incorporating the semi-synthetic promoters. Finally, utilisation of selected semi-synthetic promoters in multivalent vaccine design is currently being explored and will likely provide further insight into the utility of the herein described novel regulatory elements for coordinating balanced co-expression of multiple antigens from a single vector backbone.

## 5. Conclusions

In summary, this work establishes a framework for engineering compact semi-synthetic promoters that circumvent the packaging constraints of the adenoviral vector platform. By leveraging vector-induced signalling pathways, we identified lead candidates like cSE3 that achieve parity with established CMV promoter variants while significantly reducing the genetic footprint of the regulatory elements. The substantially reduced size, combined with reduced inter-sequence homology within the panel, potentially allows for the stable integration of multiple expression cassettes within a single vector without risking genome instability and provides a versatile foundation for next-generation multivalent vaccines.

## 6. Patents

A patent application has been filed covering the use of the semi-synthetic promoters described here.

## Data Availability

The data presented in this study is available in this article.

## Supplementary Materials

The following supporting information can be downloaded at: https://www.mdpi.com/article/10.3390/vaccines14030260/s1: Table S1. Synthetic Enhancer (SE) Sequences.

## Abbreviations

The following abbreviations are used in this manuscript:

AAV	Adeno-associated Virus
AdV	Adenovirus
ANOVA	Analysis of Variance
AP-1	Activator Protein 1
ATF	Activating Transcription Factor
ATCC	American Type Culture Collection
a.u.	Arbitrary Units
AWERB	Animal Welfare and Ethical Review Board
BAC	Bacterial Artificial Chromosome
bGH	Bovine Growth Hormone (polyadenylation signal)
CEBPB	CCAAT/Enhancer-Binding Protein Beta
ChAdOx1	Chimpanzee Adenovirus Oxford 1
CMV	Cytomegalovirus
CS	Circumsporozoite protein
CSN	Codon-optimised *Plasmodium falciparum* circumsporozoite antigen
cSE	Semi-synthetic promoter (e.g., cSE1–cSE8)
DBP	DNA-Binding Protein (adenoviral)
DMEM	Dulbecco’s Modified Eagle Medium
DRE	Dioxin-Response Element
DTT	Dithiothreitol
EGFP	Enhanced Green Fluorescent Protein
ELISA	Enzyme-Linked Immunosorbent Assay
ELISpot	Enzyme-Linked ImmunoSpot
EU	ELISA Units
FBS	Foetal Bovine Serum
GAPDH	Glyceraldehyde 3-phosphate dehydrogenase
GFP	Green Fluorescent Protein
HAdV-C5	Human Adenovirus Serotype 5
HRP	Horseradish Peroxidase
HSV	Herpes Simplex Virus
IE	Immediate-Early
IFN-γ	Interferon-gamma
IgG	Immunoglobulin G
IRF	Interferon Regulatory Factor
IUs	Infectious Units
IVC	Individually Ventilated Cage
lpCMV	Long Cytomegalovirus promoter
MFI	Mean Fluorescence Intensity
MOI	Multiplicity of Infection
NF-κB	Nuclear Factor kappa B
NF-Y	Nuclear Factor Y
Nrf2	Nuclear Factor E2-Related Factor 2
ORF	Open Reading Frame
PBMC	Peripheral Blood Mononuclear Cell
PFM	Position Frequency Matrix
PVDF	Polyvinylidene Difluoride
RT-qPCR	Quantitative Reverse Transcription Polymerase Chain Reaction
SD	Standard Deviation
SE	Synthetic Enhancer module
SEM	Standard Error of the Mean
SFC	Spot-Forming Cells
SP1	Specificity Protein 1
spCMV	Short Cytomegalovirus promoter
SPF	Specific Pathogen-Free
STAT	Signal Transducer and Activator of Transcription
TetO	Tetracycline Operator
TetR	Tetracycline Repressor
TF	Transcription Factor
TFBS	Transcription Factor Binding Site
TSS	Transcription Start Site
WT	Wild-Type
