# Supplementary material for: Development and Evaluation of Compact Semi-Synthetic Promoters for Enhanced Antigen Expression in Adenoviral-Vectored Vaccines
Source: Vaccines (Basel). 2026 Mar 13;14(3):260. doi: 10.3390/vaccines14030260 (PMC13030575; doi:10.3390/vaccines14030260)
Supplement: Supplementary file 1 [file vaccines-14-00260-s001.zip › vaccines-4156264-supplementary.pdf]

vaccines

MDPI

Supplementary

Table S1. Synthetic Enhancer (SE) Sequences

SE	Sequence 5′-3′
SE1	ACTAGAGAAACGGAAACCGAAACTATCCTGGGAATTTCCACTAGATTTCCAGGAA-<br>TATCCTGTGACTCATACAGATGGGGATTTCCAAAGGTGGGGCGGGGTTATCCTGAATTCCCC<br>ACAGATGGGGCGGGGTACTAGAGAATTCCCCACCTTAGGGGCGGGGTAAAGGTGGGGAT-<br>TTCCACCTTAGGGAATTTCCACAGAT
SE2	ACTAGAGATGACGTCATCAAAGGTGGGGATTTCCACCTTAATGACTCAGCAACCTTAGCTT-<br>GCGTGAGAAGTATCCTGGGGATTTCCACAGATGCTTGCGTGAGAAGACCTTAGAATTCCCCT<br>ATCCTATTGCACAATAAAGGTGGGAATTTCCACAGATATGACTCAGCAACTAGAGGGGAT-<br>TTCCTATCCTCAGCCAATCAG
SE3	TATCCTACTAGAGAATTCCCCTATCCTATGACTCAGCAACTA-<br>GAGGGAATTTCCAAAGGTGGGGCGGGGTACCTTAGGGGATTTCCACAGATATGACTCAGCA<br>TATCCTGAATTCCCCACCTTAGGGGCGGGGTTATCCTGGGAATTTCCACTA-<br>GAGGGGCGGGGTACAGATGGGGATTTCCACCTTAGAATTCCCCACTAGA
SE4	TATCCTAGAAACAGAAAACAGATGAAACGGAAACCGAAACTATCCTGGGGAT-<br>TTCCAAAGGTTTTCCAGGAAATATCCTGAATTCCCCACTAGATTTCCAGGAAAACAGATCGA<br>AAGTGAAAGTACCTTAGAATTCCCCACAGATAGAAACAGAAAACCTTAATGACTCAGCAT-<br>ATCCTGGAAAATGAAACTGACAGATGGGGATTTCCACTAGA
SE5	TATCCTACTAGAGAATTCCCCACAGATATGAGTCATTATCCTGGGGATTTCCAAAGGTT-<br>GTGACTCATTATCCTGAATTCCCCACTAGAATTGCACAATACCTTAGATGACGTCATCACCTT<br>AGGGAATTTCCTATCCTATGATGCAATAAAAGGTGCTTGCGTGAGAAGTATCC-<br>TATGACTCAGCAACAGATGGGGATTTCCACTAGA
SE6	AAAGGTATTGCACAATACTAGAGGGGATTTCCTATCCTATGAG-<br>TCATAAAGGTGGGGCGGGG-<br>TACCTTAGGGAATTTCCAAAGGTGATGACGTCATCACAGATCGAAAGTGAAAGTTATCCTG<br>AATTCCCCACTAGATTTCCAGGAAAACAGATGCTTGCGTGAGAAGTATCCTG-<br>GAAAATGAAACTGACTAGAGGGAATTTCCACAGAT
SE7	AAAGGTGGGAATTTCCTATCCTTTTCCAGGAAAACAGATTGTGACTCATAAAGGTG-<br>GAAAATGAAACTGACTAGAGGGGATTTCCTATCCTATGAGTCATAAAGGTGAATTCCCCAC<br>AGATATGATGCAATAACCTTACGAAAGTGAAAGTTATCCTGATGACGTCATCACCTTA-<br>GAAACGGAAACCGAAACACTAGACAGCCAATCAG
SE8	AAAGGTGGGAATTTCCACTAGAATGAGTCATTATCCTGATGACGTCATCAAAGGTGGGGAT-<br>TTCCACAGATGAAACGGAAACCGAAACTATCCTATTGCACAATACTAGAGCTTGCGTGAGA<br>AGACCTTAGAATTCCCCACTAGAGGGGCGGGGTACAGATCGAAAGTGAAAGTAC-<br>CTTAATGACTCAGCAACAGATGGGGATTTCCTATCCT

 https://doi.org/10.3390/vaccines14030260
